# At-Homeness Influences the Cognition of Multimorbid Older Adults: Longitudinal Path Analysis Through Loneliness

**DOI:** 10.1093/geroni/igab046.1466

**Published:** 2021-12-17

**Authors:** Daniel R Y Gan, John Best, Andrew Wister

**Affiliations:** Simon Fraser University, Vancouver, British Columbia, Canada

## Abstract

Approximately two-thirds of older adults’ experience multimorbidity in North America. Challenges of symptoms management and reduced mobility often coincide with late-life depression which is associated with a 2 to 5-fold increased dementia risk. Loneliness and depression are connected in the prodromal phases. We examine the effects of physical environment (e.g., housing and neighborhood factors) and social environment (e.g., social support) on loneliness, depression, and cognition using path analysis, controlling for baseline. Data(n=15,087) was drawn from the Canadian Longitudinal Study on Aging. Measures of housing, neighborhood and life satisfaction were used to construct an index of “at-homeness” based on theory. We found good model fit (TLI=.989; CFI=.999; RMSEA=.026; SRMR=.006). At-homeness(B=-.20, p<.001) rivaled the effect of social environment(B=-.19, p<.001) on loneliness. Together, physical environment and loneliness had as much effect on cognition as depression. If causality is supported, modifying older adults’ satisfaction with their home environment may reduce loneliness and cognitive decline.

